# Long-term outcomes and worse clinical course in Takotsubo syndrome patients with amyotrophic lateral sclerosis

**DOI:** 10.2459/JCM.0000000000001711

**Published:** 2025-02-28

**Authors:** Luca Fazzini, Alessandro Martis, Maria Ida Pateri, Alessandra Maccabeo, Giuseppe Borghero, Monica Puligheddu, Roberta Montisci, Maria Francesca Marchetti

**Affiliations:** a Clinical Cardiology Unit, Department of Medical Sciences and Public Health, University of Cagliari; b Department of Medical Sciences and Public Health, Institute of Neurology, University of Cagliari; c Neurology Unit, AOU Cagliari, Hospital D. Casula Monserrato, Cagliari, Italy

**Keywords:** acute heart failure, amyotrophic lateral sclerosis, echocardiography, Takotsubo syndrome

## Abstract

**Aims:**

Takotsubo syndrome (TTS) is usually triggered by either physical/psychological stressors or comorbidities, neurological among others. The prevalence of amyotrophic lateral sclerosis (ALS) among TTS and whether it has a worse clinical course is not known. We aim to describe ALS prevalence and its impact on clinical presentation, clinical course, and long-term mortality.

**Methods:**

We retrospectively screened the overall TTS population admitted and followed up at our institution between 2007 and 2020. Clinical, electrocardiographic, and echocardiographic data were collected. Kaplan–Meier method was applied for time-to-event analysis to assess the outcome of interest of all-cause death.

**Results:**

Eighty-five patients with TTS were included in our study. Overall, the mean age was 70 ± 12 years, 86% were females. Six patients (7% prevalence) were affected by ALS. At admission, patients with ALS were more likely to present left ventricular systolic dysfunction (*P* = 0.007). The clinical course of ALS patients was more likely complicated by cardiogenic shock (*P* = 0.003) which required catecholamines infusion (*P* = 0.001) and mechanical ventilation (*P* = 0.009). Despite similar in-hospital mortality rates, ALS patients exhibited significantly elevated all-cause mortality during a median 6-year follow-up (hazard ratio, 19.189, 95% confidence interval 5.639–65.296, log-rank test *P* < 0.001) with significantly shorter hospitalization to death time (*P* = 0.039).

**Conclusions:**

Our findings highlight a notable prevalence of ALS among TTS patients, with worse clinical presentation and in-hospital course in ALS-affected individuals. While in-hospital mortality rates were comparable, highlighting the reversible nature of TTS in both groups, long-term follow-up revealed significantly heightened all-cause mortality in ALS patients, emphasizing the impact of ALS on patient prognosis.

## Graphical abstract

**Figure:**
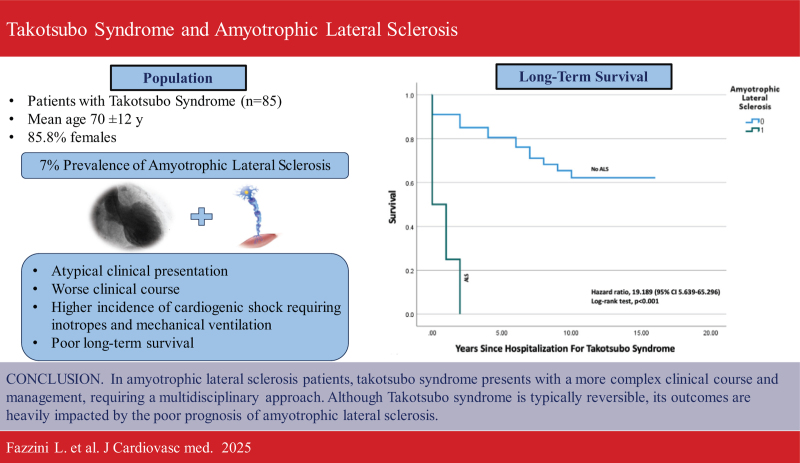

## Introduction

Takotsubo syndrome (TTS), also known as stress cardiomyopathy or apical ballooning syndrome, manifests as an acute, transient, and reversible impairment of the left ventricular function. It shares clinical resemblances with acute coronary syndromes, including comparable symptoms upon presentation, elevated cardiac biomarkers, and abnormalities in the electrocardiogram (ECG). TTS prevalence ranges from 1–3%^1,2^ to 5–6%^3,4^ of female patients presenting with ST-segment elevation. Females are much more affected than males since 90% of TTS cases are females,^5,6^ usually older than 55 years.^7^ The most common clinical presentation involves symptoms such as oppressive chest pain, dyspnea, or syncope accompanied by new ST-segment abnormalities on the ECG. This presentation poses a significant challenge in the initial assessment, closely resembling the symptoms of acute myocardial infarction and making it difficult to differentiate between the two conditions during the initial evaluation. Recently, artificial intelligence tools have been shown to play a role in supporting differential diagnosis.^8,9^

According to the InterTAK diagnostic criteria,^10^ TTS is defined by transient left ventricular dysfunction with regional wall motion abnormalities extending beyond a single epicardial vascular distribution associated with new ECG abnormalities and increased cardiac biomarkers. An emotional or physical trigger^11^ may be present including pheochromocytoma and neurologic disorders (such as subarachnoid hemorrhage). The presence of significant coronary artery disease does not exclude TTS diagnosis and infectious myocarditis must be ruled out. Traditionally, TTS has been linked to negative emotional stressors such as mourning or quarrels, as well as psycho-physical triggers like the exacerbation of an illness or the diagnosis of an acute condition.^12^ However, approximately 4% of emotionally triggered TTS are associated with positive emotions and joyful events, colloquially referred to as ‘happy heart syndrome’.^13,14^ In some instances, no specific trigger can be identified.^12^ Additionally, individuals hospitalized for TTS often present with multiple comorbidities, particularly of neurological, psychiatric, and autoimmune nature.^12,15,16,17^

Amyotrophic lateral sclerosis (ALS) is a heterogeneous neurodegenerative syndrome, also known as motor neuron disease, characterized by muscle weakness and paralysis due to degeneration of both upper and lower motor neurons which leads to the end-stage disease marked by respiratory failure.^18^ The etiology of ALS is unknown. Some affected individuals present familiar forms given by gene mutations with a wide range of functions, even functions in nonmotor cells.^18^

While the association between ALS and TTS has been documented in the literature, it remains poorly understood. We hypothesized that TTS might be a prevalent complication in ALS patients, and those with both ALS and TTS could experience a more unfavorable clinical trajectory, potentially leading to deterioration and eventual mortality attributed to undiagnosed stress cardiomyopathy. This study aimed to determine the prevalence of ALS in patients affected by TTS and to investigate differences in clinical course and long-term mortality.

## Methods

### Study population

This is a single-center, observational, retrospective study. We retrospectively analyzed 85 patients with TTS admitted to our Intensive Cardiology Unit between September 2007 and October 2020. TTS diagnosis was made according to the criteria of the International Expert Consensus Document on TTS.^10^ Accordingly, all patients underwent coronary angiography to exclude a coronary artery disease (defined as stenosis >50%) and had to fit the previously mentioned criteria before inclusion. We collected the clinical characteristics, risk factors, circadian and seasonal rhythm of the clinical presentation, electrocardiographic presentation, blood exams, therapy during hospitalization and discharge, adverse events during hospitalization, and follow-up. The population was divided into two groups based on the presence of ALS diagnosis.

### Electrocardiogram

The 12-lead Standard ECG plus right and posterior leads were recorded at admission, at the end of the coronary angiography, and every 24 h for the following 4 days. All patients were monitored 24 h a day for arrhythmic events. The duration of the QT interval was determined in D2 as the average of three different RR intervals and the correct QT (QTc) was then calculated according to Bazzet's formula.

### Echocardiogram

All patients underwent standard mono-bidimensional and color Doppler echocardiography which echocardiography expert cardiologists performed at both admission and discharge. All measurements were made three times in patients with sinus rhythm, while in those with atrial fibrillation, we averaged measurements over 10 cycles, according to the recommendations of the European/American Society of Echocardiography.^19^ We collected end-diastolic diameters (EDD) measured with B-mode, end-diastolic volumes (EDV), end-systolic volumes (ESV), left ventricular ejection fraction (LVEF) derived from biplane Simpson's method, and wall motion score index (WMSI). The left ventricle was therefore divided into 16 segments and each segment was analyzed according to its motility and thickening as follows: normal or hyperkinetic, hypokinetic, akinetic, dyskinetic/aneurysmal.

### Clinical follow-up and outcome

Between September 2007 and January 2024, data on all-cause mortality events were systematically collected. Inpatient events were documented by on-site medical professionals, while outpatient events were remotely recorded by two investigators who are also included among the authors (M.F.M., A.M.).

### Statistical analysis

Continuous variables are expressed as mean ± standard deviation or median, and categorical data as percentages. Continuous variables were analyzed with the Student's *t*-test for paired and unpaired data and the Kruskal–Wallis test for nonparametric data. Chi-squared or Fisher's exact test was used to analyze the categorical variables. Kaplan–Meyer curves were generated and compared using a log-rank test, and hazard ratios were evaluated using the Cox proportional hazards model. A value of *P* < 0.05 was considered significant. The data were analyzed with the SPSS software, version 22.0 (SPSS, Inc., Chicago, IL).

## Results

Eighty-five patients with TTS diagnosis were included. Table 1 summarizes the baseline characteristics. The mean age was 70 ± 12 years (range 44–96) with a predominance of female patients (85.8%). Notably, 96.5% of the total population were aged over 50 years. Several comorbidities including hypothyroidism due to Hashimoto's thyroiditis (10.5%), hyperthyroidism (3.5%), psychiatric disorders (17.6%), and neurological diseases (14.1%) were identified. Among the patients with neurological disorders, six of them (7% overall) were affected by ALS. Overall, most of them presented with typical chest pain (63.5%) and had a history of a recent stressful event (64.7%) encompassing both emotional and physical stressors. Acute pulmonary edema was observed in 10 patients. Approximately half of the population exhibited ST-segment elevation on the ECG. Concerning echocardiographic features, 18% (*n* = 15) had LVEF below 35% at admission. Overall, the mean LVEF was 46.1 ± 10.6% and the WMSI was 1.89 ± 0.32 (Table 2). Sixteen patients presented with left ventricular outflow tract obstruction.

**Table 1:** Baseline characteristics of patients with Takotsubo syndrome according to the presence of amyotrophic lateral sclerosis

	Overall (*n* = 85)	ALS (*n* = 6)	No ALS (*n* = 79)	*P*-value
Age (years)	70 ± 12	74 ± 4	70 ± 12	0.090
Female, *n* (%)	73 (85.8%)	6 (100%)	67 (84.8%)	0.300
Hypercholesterolemia, *n* (%)	37 (43.5%)	0 (0%)	37 (46.8%)	0.026
Hypertension, *n* (%)	51 (60%)	6 (100%)	45 (57)	0.038
Obesity, *n* (%)	5 (5.9%)	0 (8%)	5 (6.3%)	0.520
Smoking, *n* (%)	12 (14.1%)	1 (16.7%)	11 (13.9%)	0.850
Diabetes, *n* (%)	14 (16.5%)	1 (16.7%)	13 (16.5%)	0.980
Coronary artery disease, *n* (%)	19 (22.3%)	3 (50%)	16 (25.3%)	0.090
Clinical presentation				
Typical chest pain, *n* (%)	54 (63.5%)	1 (16.7%)	53 (67.1%)	0.038
Atypical chest pain, *n* (%)	5 (6%)	2 (33.3%)	3 (3.7%)	0.003
Dyspnea, *n* (%)	29 (34.5%)	3 (50%)	26 (33.3)	0.34
Stressful event, *n* (%)	55 (64.7%)	4 (66.7%)	51 (64.6%)	0.910
Emotional trigger, *n* (%)	20 (34.1%)	1 (16.7%)	28 (35.4%)	0.350
Physical trigger, *n* (%)	26 (30.6%)	3 (50%)	23 (29.1%)	0.280
Pulmonary edema, *n* (%)	10 (11.8%)	2 (33.3%)	8 (10.1%)	0.080
Inverted T waves, *n* (%)	29 (34.1)	3 (50%)	26 (35.6%)	0.480
ST-segment elevation, *n* (%)	40 (47%)	2 (33.3%)	38 (52.1)	0.370
ST-segment depression, *n* (%)	3 (3.5%)	0 (0%)	3 (4.1%)	0.610
Long QTc, *n* (%)	19 (26.9%)	2 (33.3%)	17 (21.5%)	0.710

ALS, amyotrophic lateral sclerosis.

**Table 2:** Baseline and discharge echocardiographic characteristics

Baseline	Overall (*n* = 85)	ALS (*n* = 6)	No ALS (*n* = 79)	*P*-value
LVEF ≤35%, *n* (%)	15 (18.1%)	3 (50%)	12 (15.6%)	0.035
LVEF (%)	46.1 ± 10.6^∗^	35.5 ± 6.8^^^	47 ± 10.4^∗^	0.007
WMSI	1.89 ± 0.32^∗∗^	2.04 ± 0.29^$^	1.88 ± 0.33^∗∗^	0.250
Apical ballooning, *n* (%)	82 (96)	6 (100)	76 (96)	0.801
Midventricular ballooning, *n* (%)	11 (13)	2 (33)	9 (11)	0.171
Severe MR, *n* (%)	1 (1)	0 (0)	1 (1)	0.929
RV dysfunction, *n* (%)	0 (0)	0 (0)	0 (0)	-
LVOTO, *n* (%)	16 (19)	2 (33)	14 (18)	0.332
LV thrombus, *n* (%)	2 (2)	0 (0)	2 (3)	0.858
At discharge				
LVEF	60.2 ± 7.4^∗^	53.1 ± 12.7^	60.8 ± 6.7^∗^	0.014
WMSI	1.21 ± 0.36^∗∗^	1.35 ± 0.29$	1.2 ± 0.36^∗∗^	0.290

^∗^*P* = 0.0001 EF at admission compared with discharge. ^∗∗^*P* = 0.0001 WMSI at admission compared with discharge. ^*P* = 0.018 EF at admission compared with discharge. $ = 0.002 WMSI at admission compared with discharge. ALS, amyotrophic lateral sclerosis; LV, left ventricular; LVEF, left ventricular ejection fraction; LVOTO, left ventricular outflow tract obstruction; MR, mitral regurgitation; RV, right ventricle; WMSI, wall motion score index.

Cardiogenic shock complicated the clinical course in five cases, necessitating inotropic/vasopressor support (3.5%) after having excluded left ventricular outflow tract obstruction and mechanical ventilation (7.1%) (Table 3). Notably, within our study population, a single in-hospital death was observed.

**Table 3:** In-hospital course

In-hospital course	Overall (*n* = 85)	ALS (*n* = 6)	No ALS (*n* = 79)	*P*-value
Cardiogenic shock, *n* (%)	5 (5.9%)	2 (33.3%)	3 (3.8%)	0.003
Catecholamines, *n* (%)	3 (3.5%)	2 (33.3%)	1 (1.2%)	0.001
Mechanical ventilation, *n* (%)	6 (7.1%)	2 (33.3%)	4 (5%)	0.009
In-hospital death, *n* (%)	1 (1.2%)	0	1 (1.3%)	0.780

ALS, amyotrophic lateral sclerosis.

### Comparison between groups stratified by amyotrophic lateral sclerosis

The mean time from ALS diagnosis to hospitalization for TTS was 13.1 ± 6.5 months. The characteristics of the two groups (affected and not affected by ALS) are shown in Table 1. Overall, ALS patients were all females with a mean age of 74 ± 4 years at admission. All the patients with ALS were affected by arterial hypertension (*P* = 0.038), while none of them was affected by hypercholesterolemia (*P* = 0.026). ALS patients less frequently presented with typical chest pain (*P* = 0.038) and two of them (33,3%) presented with atypical chest pain compared with the control group which was more likely characterized by a typical presentation (*P* = 0.003). We found no differences in smoking diabetes, obesity, coronary artery disease, physical or emotional triggers, ECG, and echocardiographic pattern presentation.

The clinical presentation among ALS patients demonstrated a more compromised state, manifesting as a greater impairment of systolic function as indicated by LVEF (35.3 ± 6.8% vs. 47 ± 10.4%, *P* = 0.007). Severe systolic dysfunction was notably present in 50% of ALS patients (*P* = 0.035). No significant differences were found in the prevalence of left ventricular outflow tract obstruction or apical and midventricular ballooning pattern presentation. None of the patients presented with right ventricular dysfunction. Upon discharge, the ALS group exhibited a significant improvement in LVEF compared with admission (35.5 ± 6.8% to 53.1 ± 12.7%; *P* = 0.018), yet it remained significantly lower than the control group (*P* = 0.014) (Table 2). Discharge therapy was largely comparable between the two groups, except for statins, which were not prescribed in the ALS group due to the absence of hypercholesterolemia in any patient.

### In-hospital outcomes and long-term follow-up

Patients in the ALS group experienced a more complicated in-hospital clinical course. Two patients (33.3%) in the ALS group presented with a cardiogenic shock (*P* = 0.003) which required catecholamines infusion (*P* = 0.001). Furthermore, patients with ALS presented with a higher incidence of acute respiratory failure for which they underwent assisted ventilation more frequently than other patients (*P* = 0.009). We did not find any differences in in-hospital mortality between the two groups.

**Fig. 1:**
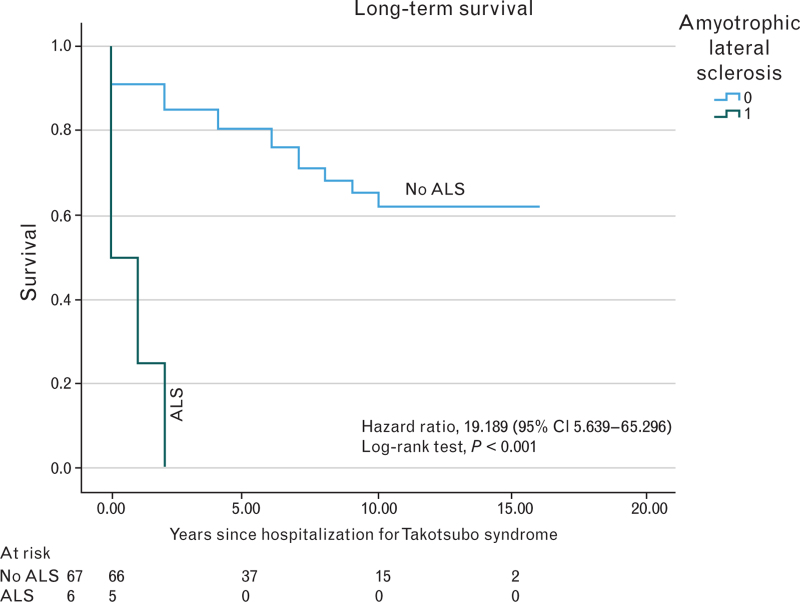
Kaplan–Meyer curves stratified by the presence of amyotrophic lateral sclerosis. Cumulative incidence of all-cause mortality among patients with Takotsubo syndrome stratified by the presence of amyotrophic lateral sclerosis. Amyotrophic lateral sclerosis was associated with increased rates of mortality up (HR, 19.189, 95% CI 5.639–65.296, log-rank test *P* < 0.001). Kaplan–Meyer curves were generated and compared using a log-rank test, and hazard ratios were evaluated using the Cox proportional hazards model. A value of *P* < 0.05 was considered significant.

In our cohort, 73 patients (86%) were followed up for a median of 6.3 years (4.2–10.4). ALS patients experienced notably higher all-cause mortality during this extended follow-up period [hazard ratio (HR) 19.189, 95% confidence interval (CI) 5.639–65.296, log-rank test *P* < 0.001] (Table 4, Fig. 1). Specifically, among the ALS subgroup, five patients died, while the sixth did not at the time of follow-up censoring. The median time from TTS hospitalization to death was significantly shorter in the ALS cohort (5.1 vs. 42.7 months, *P* = 0.039).

**Table 4:** Long-term mortality

	Death/*N*	Effect	
		HR (95% CI)	Log-rank test
Group			
No ALS	20/67 (29.6%)	1.00 (Ref.)	
ALS	5/6 (83.3%)	HR 19.189 (95% CI 5.639–65.296)	<0.001

ALS, amyotrophic lateral sclerosis; CI, confidence interval; HR, hazard ratio.

## Discussion

This retrospective study was designed to explore the prevalence of ALS in patients hospitalized with TTS. The primary objective was to compare the clinical presentation, hospitalization course, and long-term mortality between TTS patients with and without ALS. The key findings of our analysis are as follows: a notable prevalence of previously diagnosed ALS among TTS patients; patients with ALS exhibited a more severe and complicated clinical presentation and in-hospital course; over a long-term outcome follow-up, patients with ALS demonstrated significantly higher mortality rates.

The association between central nervous system disorders and cardiac manifestations has been extensively documented.^20^ Conditions affecting the brain and heart may manifest through various cardiovascular issues, including arrhythmias, arterial hypertension, myocardial infarction, heart failure, and, notably, TTS. Among central nervous system disorders, subarachnoid hemorrhage,^21,22^ epilepsy,^23^ and stroke^24,25^ have been identified as potential triggers for TTS. In the context of ALS, only a limited number of TTS cases have been reported in published cohorts. Specifically, Izumi *et al.* and Choi *et al.* reported a prevalence of TTS of 1.6% and 1.4% respectively, within populations affected by ALS.^26,27^ Precisely, the cohort studied by Choi *et al.* observed an increase in the prevalence to 14% when considering only ALS patients who underwent echocardiogram for several reasons.^27^ Our analysis, combined with these prevalence data, highlights the necessity for screening for acute cardiac dysfunction in ALS patients experiencing sudden clinical deterioration and respiratory failure. This is crucial, as several stress cardiomyopathies may have gone undetected and unrecognized. Moreover, ALS patients unavoidably experience progressive motor neuron degeneration, culminating in respiratory failure and eventual mortality.^18,28^ Considering our results, we hypothesized that some patients who died of respiratory failure might have been affected by an undiagnosed TTS. Specifically in our population, one patient presented with TTS immediately after placement of percutaneous endoscopic gastrostomy for parenteral nutrition, and three patients during bronchopneumonia. Therefore, in the case of acute or potentially stressful events, it is advisable to work out an ECG and echocardiogram, in order to screen acute myocardial dysfunction. A potential physio-pathological rationale for our observed significant association between TTS and ALS is the persistent cardiac sympathetic hyperactivity experienced by ALS patients. This hyperactivity has been linked to both sudden cardiac death and stress-induced cardiomyopathy.^29^ Additionally, autonomic system disorders further complicate the course of ALS, elevating the risk of sudden death.^30^

Our findings revealed notable discrepancies compared with earlier reports.^26,27^ Firstly, our ALS patients were significantly older, with an average age of 74 years, in comparison with the populations from previously referenced cohorts (61 and 65 years). Secondly, the median time between ALS diagnosis and TTS presentation was 13 months, with 50% of patients developing TTS within 1 year of ALS diagnosis. This observation apparently contrasts with existing literature, in which TTS is often perceived as a complication in the late phase or end stage of the disease. However, despite the recent diagnosis, all the patients presented with TTS while having an ALSFRS-R score below 25, thus in an advanced stage of the disease.^31^

Concerning clinical presentation, the onset of TTS in ALS patients is frequently characterized by atypical chest pain and dyspnea.^26,27,32,33^ The presence of atypical symptoms, often associated with difficulty in speaking in ALS patients, may contribute to the underdiagnosis of TTS in this specific population. Additionally, the ALS group exhibited a more adverse in-hospital course, with 33% of patients complicated by cardiogenic shock requiring intravenous inotropes/vasopressors and mechanical ventilation postorotracheal intubation, compared with 5.1% of the group without ALS. Notably, upon admission, none of the ALS patients presented with severe chronic respiratory failure or required ventilatory support or tracheostomy. However, the inherent muscle impairment in ALS patients may contribute to the onset of acute respiratory failure, complicating the course of TTS. Moreover, the ALS group demonstrated significantly poorer cardiac function at admission than the control group, as evidenced by the reduced mean LVEF. Although ALS patients showed recovery during hospitalization, only 50% achieved complete normalization of cardiac function, contrasting with 88% in the control group. Consequently, patients with ALS and TTS may warrant a more intensive cardiological follow-up after discharge.

Despite the more complicated clinical course in the ALS population, we did not report any differences in terms of in-hospital mortality. This observation underscores the reversible nature of TTS in both subgroups, as it represents a treatable cause of ventricular dysfunction. However, five ALS patients experienced significantly earlier mortality compared with the control group, emphasizing the influence of ALS on long-term prognosis.^34,35^

Our study has inherent limitations associated with its design. Firstly, the data are derived from a retrospective observational approach, introducing the possibility of biases inherent to such study designs. Secondly, given its monocentric nature, the sample size is limited; nonetheless, it remains noteworthy when compared with the sample sizes in major studies already published within this clinical context. Nevertheless, the generalizability of our findings warrants confirmation through larger patient cohorts. Thirdly, our study does not provide an explanation for the pathophysiological mechanism underlying TTS in patients with ALS. This calls for dedicated investigations specifically designed to analyze the role of the heart–brain axis, and further research could elucidate this phenomenon.

## Conclusion

In conclusion, our findings underscore the significant link between ALS and TTS, providing valuable insights for clinicians to identify ALS as a potential predisposing factor for TTS. Given the frequent occurrence of triggers and precipitating factors, both emotional and physical (such as surgery, respiratory distress, or infection) in these patients, a comprehensive screening for TTS is essential. When TTS presents in ALS patients, the clinical management becomes notably more complex, necessitating a multidisciplinary approach. Moreover, while in-hospital mortality rates were comparable across groups, individuals with ALS and concurrent TTS require diligent cardiological and neurological follow-up due to their elevated mortality risk likely influenced by the prognosis of ALS.

## Acknowledgements

None.

All authors take responsibility for all aspects of the reliability and freedom from bias of the data presented and their discussed interpretation.

Funding: This research did not receive any specific grant from funding agencies in the public, commercial, or not-for-profit sectors.

### Conflicts of interest

There are no conflicts of interest.

## References

[1] Prasad A Dangas G Srinivasan M. Incidence and angiographic characteristics of patients with apical ballooning syndrome (takotsubo/stress cardiomyopathy) in the HORIZONS-AMI trial: an analysis from a multicenter, international study of ST-elevation myocardial infarction. *Catheter Cardiovasc Interv* 2014; 83:343–348. 22121008 10.1002/ccd.23441

[2] Bybee KA Prasad A Barsness GW. Clinical characteristics and thrombolysis in myocardial infarction frame counts in women with transient left ventricular apical ballooning syndrome. *Am J Cardiol* 2004; 94:343–346. 15276100 10.1016/j.amjcard.2004.04.030

[3] Redfors B Vedad R Angerås O. Mortality in takotsubo syndrome is similar to mortality in myocardial infarction – a report from the SWEDEHEART registry. *Int J Cardiol* 2015; 185:282–289. 25818540 10.1016/j.ijcard.2015.03.162

[4] Arcari L Núñez-Gil I Stiermaier T. Gender differences in Takotsubo syndrome. *J Am Coll Cardiol* 2022; 79:2085–2093. 35618345 10.1016/S0735-1097(22)03076-5 PMC8972425

[5] Templin C Ghadri JR Diekmann J. Clinical features and outcomes of Takotsubo (stress) cardiomyopathy. *N Engl J Med* 2015; 373:929–938. 26332547 10.1056/NEJMoa1406761

[6] Schneider B Athanasiadis A Stöllberger C. Gender differences in the manifestation of Tako-tsubo cardiomyopathy. *Int J Cardiol* 2013; 166:584–588. 22192296 10.1016/j.ijcard.2011.11.027

[7] Deshmukh A Kumar G Pant S. Prevalence of Takotsubo cardiomyopathy in the United States. *Am Heart J* 2012; 164:66–71e1. 22795284 10.1016/j.ahj.2012.03.020

[8] Laumer F Di Vece D Cammann VL. Assessment of artificial intelligence in echocardiography diagnostics in differentiating Takotsubo syndrome from myocardial infarction. *JAMA Cardiol* 2022; 7:494–503. 35353118 10.1001/jamacardio.2022.0183 PMC8968683

[9] De Filippo O Cammann VL Pancotti C. Machine learning-based prediction of in-hospital death for patients with Takotsubo syndrome: the InterTAK-ML model. *Eur J Heart Fail* 2023; 25:2299–2311. 37522520 10.1002/ejhf.2983

[10] Ghadri JR Wittstein IS Prasad A. International Expert Consensus Document on Takotsubo Syndrome (Part II): diagnostic workup, outcome, and management. *Eur Heart J* 2018; 39:2047–2062. 29850820 10.1093/eurheartj/ehy077 PMC5991205

[11] Pätz T Santoro F Cetera R. Trigger-associated clinical implications and outcomes in Takotsubo syndrome: results from the multicenter GEIST Registry. *J Am Heart Assoc* 2023; 12:e028511. 37421264 10.1161/JAHA.122.028511 PMC10382102

[12] Lyon AR Citro R Schneider B. Pathophysiology of Takotsubo syndrome: JACC State-of-the-Art Review. *J Am Coll Cardiol* 2021; 77:902–921. 33602474 10.1016/j.jacc.2020.10.060

[13] Stiermaier T Walliser A El-Battrawy I. Happy heart syndrome: frequency, characteristics, and outcome of Takotsubo syndrome triggered by positive life events. *JACC Heart Fail* 2022; 10:459–466. 35772855 10.1016/j.jchf.2022.02.015

[14] Fazzini L Marchetti MF Biddau M. The happiness for Italy's victory at the European soccer championships costs a ‘happy heart syndrome’. *Eur J Case Rep Intern Med* 2022; 9:003572. 36506735 10.12890/2022_003572 PMC9728223

[15] Sancassiani F Carta MG Montisci R. Takotsubo syndrome is associated with mood disorders and antidepressants use, not with anxiety and impairment of quality of life due to the psychiatric disorder. *Clin Pract Epidemiol Ment Health* 2018; 14:26–32. 29541148 10.2174/1745017901814010026 PMC5838620

[16] Alim S Shah H Zahera SM Rahmatova J Irfan M Mahmood Z Zahra SA. An update on Takotsubo syndrome. *J Cardiovasc Med (Hagerstown)* 2023; 24 (10):691–699. 37577868 10.2459/JCM.0000000000001528

[17] Rosh B Naoum I Stein N Jaffe R Saliba W. Trends in occurrence of takotsubo syndrome and association with SARS-CoV-2 infection and COVID-19 vaccination. *J Cardiovasc Med (Hagerstown)* 2023; 24 (11):815–821. 37577873 10.2459/JCM.0000000000001541

[18] Brown RH Al-Chalabi A. Amyotrophic lateral sclerosis. *N Engl J Med* 2017; 377:162–172. 28700839 10.1056/NEJMra1603471

[19] Lang RM Badano LP Mor-Avi V. Recommendations for cardiac chamber quantification by echocardiography in adults: an update from the American Society of Echocardiography and the European Association of Cardiovascular Imaging. *Eur Heart J Cardiovasc Imaging* 2015; 16:233–270. 25712077 10.1093/ehjci/jev014

[20] Finsterer J Wahbi K. CNS disease triggering Takotsubo stress cardiomyopathy. *Int J Cardiol* 2014; 177:322–329. 25213573 10.1016/j.ijcard.2014.08.101

[21] Shimada M Rose JD. Takotsubo cardiomyopathy secondary to intracranial hemorrhage. *Int J Emerg Med* 2014; 7:33. 25635193 10.1186/s12245-014-0033-4 PMC4306060

[22] Kilbourn KJ Levy S Staff I. Clinical characteristics and outcomes of neurogenic stress cardiomyopathy in aneurysmal subarachnoid hemorrhage. *Clin Neurol Neurosurg* 2013; 115:909–914. 23021080 10.1016/j.clineuro.2012.09.006

[23] Stöllberger C Wegner C Finsterer J. Seizure-associated Takotsubo cardiomyopathy. *Epilepsia* 2011; 52:e160–e167. 21777230 10.1111/j.1528-1167.2011.03185.x

[24] Jung JM Kim JG Kim JB. Takotsubo-like myocardial dysfunction in ischemic stroke: a hospital-based registry and systematic literature review. *Stroke* 2016; 47:2729–2736. 27729583 10.1161/STROKEAHA.116.014304

[25] Yoshimura S Toyoda K Ohara T. Takotsubo cardiomyopathy in acute ischemic stroke. *Ann Neurol* 2008; 64:547–554. 18688801 10.1002/ana.21459

[26] Izumi Y Miyamoto R Fujita K. Distinct incidence of Takotsubo syndrome between amyotrophic lateral sclerosis and synucleinopathies: a cohort study. *Front Neurol* 2018; 9:1099. 30619056 10.3389/fneur.2018.01099 PMC6300466

[27] Choi SJ Hong YH Shin JY. Takotsubo cardiomyopathy in amyotrophic lateral sclerosis. *J Neurol Sci* 2017; 375:289–293. 28320151 10.1016/j.jns.2017.02.012

[28] Kiernan MC Vucic S Cheah BC. Amyotrophic lateral sclerosis. *Lancet* 2011; 377:942–955. 21296405 10.1016/S0140-6736(10)61156-7

[29] Tanaka Y Yamada M Koumura A. Cardiac sympathetic function in the patients with amyotrophic lateral sclerosis: analysis using cardiac [123I] MIBG scintigraphy. *J Neurol* 2013; 260:2380–2386. 23784610 10.1007/s00415-013-7005-0

[30] Asai H Hirano M Udaka F. Sympathetic disturbances increase risk of sudden cardiac arrest in sporadic ALS. *J Neurol Sci* 2007; 254. 10.1016/j.jns.2007.01.007 17303172

[31] Cedarbaum JM Stambler N Malta E. The ALSFRS-R: a revised ALS functional rating scale that incorporates assessments of respiratory function. BDNF ALS Study Group (Phase III). *J Neurol Sci* 1999; 169:13–21. 10540002 10.1016/s0022-510x(99)00210-5

[32] Matsuyama Y Sasagasako N Koike A. An autopsy case of amyotrophic lateral sclerosis with ampulla cardiomyopathy. *Rinsho Shinkeigaku* 2008; 48:249–254. 18453156 10.5692/clinicalneurol.48.249

[33] Suzuki Y Oishi M Kanno A. Amyotrophic lateral sclerosis accompanying elevated catecholamines occurring as a complication of takotsubo cardiomyopathy. *Geriatr Gerontol Int* 2013; 13:240–241. 23286571 10.1111/j.1447-0594.2012.00943.x

[34] Chiò A Logroscino G Hardiman O. Eurals Consortium Prognostic factors in ALS: a critical review. *Amyotroph Lateral Scler* 2009; 10:310–323. 19922118 10.3109/17482960802566824 PMC3515205

[35] Traxinger K Kelly C Johnson BA. Prognosis and epidemiology of amyotrophic lateral sclerosis: analysis of a clinic population, 1997–2011. *Neurol Clin Pract* 2013; 3:313–320. 24195020 10.1212/CPJ.0b013e3182a1b8ab PMC3787117

